# METTL3-mediated m6A methylation of C1qA regulates the Rituximab resistance of diffuse large B-cell lymphoma cells

**DOI:** 10.1038/s41420-023-01698-2

**Published:** 2023-11-01

**Authors:** Junping Li, Zhigang Zhu, Yuan Zhu, Jinqing Li, Kangbao Li, Weijie Zhong

**Affiliations:** 1 Department of Geriatrics, Hematology & Oncology Ward, the Second Affiliated Hospital, School of Medicine, South China University of Technology, 510180 Guangzhou, Guangdong China; 2 Department of Geriatrics, Gastroenterology Ward, the Second Affiliated Hospital, School of Medicine, South China University of Technology, 510180 Guangzhou, Guangdong China

**Keywords:** Tumour-suppressor proteins, Transcriptional regulatory elements, Cancer therapeutic resistance

## Abstract

Rituximab has been incorporated into the standard treatment regimen for diffuse large B-cell lymphoma (DLBCL), and induces the death of tumor cells via complement-dependent cytotoxicity (CDC). Unfortunately, the resistance of DLBCL cells to Rituximab limits its clinical usefulness. It remains unclear whether the complement system is related to Rituximab resistance in DLBCL. A Rituximab-resistant DLBCL cell line (Farage/R) was generated under the stress of Rituximab. Constituent proteins of the complement system in wild-type Farage cells (Farage/S) and Farage/R cells were analyzed by qPCR, western blotting, and immunofluorescence. In vitro and in vivo knockdown and overexpression studies confirmed that the complement 1Q subcomponent A chain (C1qA) was a regulator of Rituximab resistance. Finally, the mechanism by which C1qA is regulated by m^6^A methylation was explored. The reader and writer were identified by pull-down studies and RIP-qPCR. Activity of the complement system in Farage/R cells was suppressed. C1qA expression was reduced in Farage/R cells due to post-transcriptional regulation. Furthermore, in vitro and in vivo results showed that C1qA knockdown in Farage/S cells decreased their sensitivity to Rituximab, and C1qA overexpression in Farage/R cells attenuated the Rituximab resistance of those cells. Moreover, METTL3 and YTHDF2 were proven to be the reader and writer for m^6^A methylation of C1qA, respectively. Knockdown of METTL3 or YTHDF2 in Farage/R cells up-regulated C1qA expression and reduced their resistance to Rituximab. In summary, the aberrant downregulation of C1qA was related to Rituximab resistance in DLBCL cells, and C1qA was found to be regulated by METTL3- and YTHDF2-mediated m6A methylation. Enhancing the response of the complement system via regulation of C1qA might be an effective strategy for inhibiting Rituximab resistance in DLBCL.

## Highlights

- The complement system was inactive in Rituximab-resistant DLBCL cells.
- The complement 1Q subcomponent A chain (C1qA) was investigated as a potential regulator of Rituximab resistance in DLBCL.
- METTL3 and YTHDF2 mediated the m6A methylation of C1qA mRNA in DLBCL cells.

## Introduction

Rituximab has been incorporated into the standard treatment regimen for diffuse large B-cell lymphoma (DLBCL), which is the most common type of lymphoma [1]. Rituximab is a chimeric type I monoclonal antibody targeting CD20. After binding to CD20, Rituximab can kill cells by 4 different methods, including induction of complement-dependent cytotoxicity (CDC), antibody-dependent cellular cytotoxicity (ADCC), antibody-dependent phagocytosis (ADP), and apoptotic activity [2]. Although Rituximab has shown promising results in treatment of DLBCL, ~50% of patients do not initially respond to Rituximab. Furthermore, many patients develop resistance to Rituximab during treatment for DLBCL [3].

The complement system, a cascade of proteases, is an important part of the innate immune system, as it can identify and eliminate foreign pathogens and host apoptotic cells. The complement system consists of three main pathways: the classical pathway, initiated by formatting of antigen-antibody complexes; the alternative pathway, initiated by permissive surfaces; and the lectin pathway, initiated by the recognition of sugar residues [4]. Activation of the complement system occurs in three stages: recognition, activation, and membrane attack. Complement 1 (C1, including C1q, C1r, and C1s) is the recognition unit. C2, C3, and C4 are the activation units. C5, C6, C7, C8, and C9 are the membrane attack units. These three activation pathways lead to the activation of C3, and a subsequent cleavage of C3 into bioactive fragments C3a (an anaphylatoxin) and C3b (an opsonin) by the C3 convertase complex. C3b can bind to the surface of a target pathogen or stressed cell to trigger phagocytosis via the membrane attack complex (MAC), consisting of C5b, C6, C7, C8, and C9 [5]. The complement system is strictly and exactly controlled by multiple regulators to protect normal cells. Any dysregulation of the complement system can induce abnormal inflammation and an immune response, which can lead to autoimmune diseases and various cancers [4, 6]. For example, the levels of C1q in the serum of glioma patients were found to be significantly up-regulated when compared to those in healthy control subjects [7]. In cancers, the complement system can regulate the tumor microenvironment, tumor immunity, and affect the efficacy of immunotherapy [8, 9]. Rituximab is a monoclonal antibody used for immunotherapy of DLBCL, and kills lymphoma cells via CDC. After binding to CD20, Rituximab exposes its Fc region and combines with C1q, thereby activating the classical complement cascade and inducing cytolysis [10]. Destruction of a complement system by treatment with cobra venom factor was found to reduce the antitumor activity of Rituximab [11, 12]. In addition, complement system activity is suppressed in Rituximab-resistant B-cell NHL cells [13]. Collectively, the above studies suggest that the complement system is related to cellular resistance to Rituximab. However, the mechanism for that resistance requires further study.

N^6^-methyladenosine (m^6^A) modification is the most common modification for eukaryotic RNAs. It largely occurs in the RRACH sequences ([G/A/U][G/A]m^6^AC[U/A/C]) within long exons, 3′-untranslated regions (3’-UTR), and near stop codons [14]. It can affect almost every stage of RNA metabolism, and thus regulate the expression of various genes. Emerging evidence shows that m^6^A methylation participates in many processes, including embryonic development, sex determination, circadian rhythm, stress responses, and tumorigenesis [15]. M^6^A methylation is a reversible reaction regulated by readers, writers, and erasers. The readers include YT521-B homology (YTH) domain-containing proteins, such as YTHDF1/2 3 and YTHDC1/2. They are responsible for identifying m^6^A modification sites and or the outcomes of m^6^A-modified mRNAs. Writers are multiprotein complexes containing METTL3, METTL14, and WTAP, which catalyze m^6^A methylation. Erasers, including FTO and ALKBH5, act as demethylases that remove m^6^A methylation from RNA [16]. Therefore, the dynamics of m^6^A methylation, controlled by readers, writers, and erasers, are important for the maintenance of normal cellular function. Several studies have revealed that dysregulation of m^6^A methylation is correlated with tumorigenesis, progression, and the drug resistance of multiple cancers, including lymphoma [17–19]. In our study, we found that m^6^A methylation affected Rituximab resistance and tumor growth by regulating the complement system in DLBCL.

## Results

### The complement system was inhibited in Rituximab-resistant DLBCL cells

**Fig. 1:**
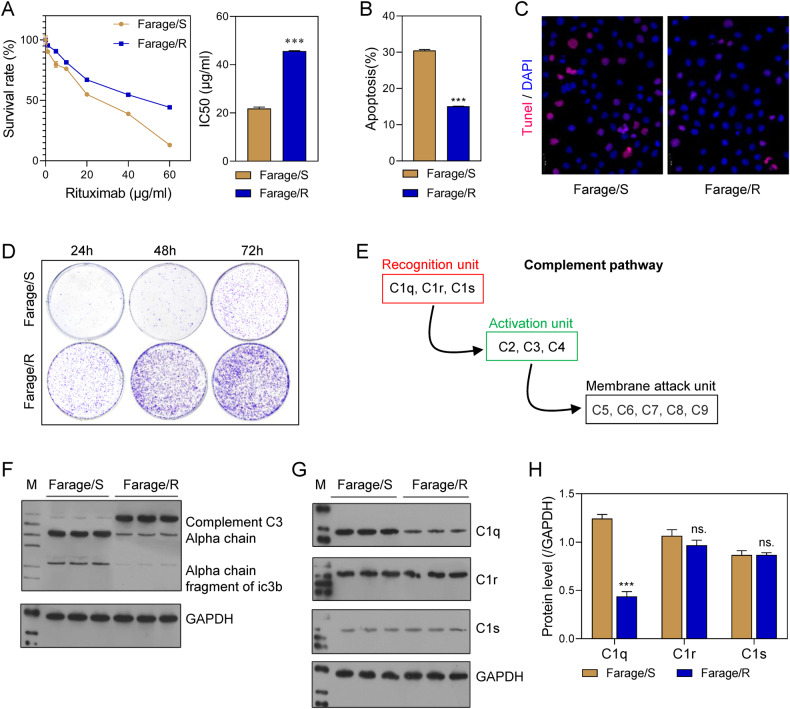
The complement system was related to Rituximab resistance in Farage cells. **A** The survival rates of Rituximab-sensitive Farage (Farage/S) and Rituximab-resistant Farage (Farage/R) cells at different concentrations of Rituximab, and the IC50 value. **B**, **C** Apoptotic cells during treatment with 20 μg/mL Rituximab were detected by flow cytometry and TUNEL staining. **D** The proliferation of cells treated with 20 μg/mL Rituximab was detected by the colony formation assay. **E** Members of the complement pathway. **F** C3 chains expressed during treatment with 20 μg/mL Rituximab were detected by western blotting. **G**, **H** Recognition units of the complement system (C1q, C1r, and C1s), were detected by western blotting. ns, not significant; ***p < 0.001.

In order to explore the role of the complement pathway in the resistance of DLBCL to Rituximab, we established a line of Rituximab-resistant DLBCL cells (Farage/R). When compared to wild-type Farage cells (Farage/S), the Farage/R cells had a higher survival rate when treated with different concentrations (1, 5, 10, 20, and 40 μg/mL) of Rituximab, and also had significantly higher IC50 value (Fig. 1A). TUNEL and flow cytometry assays revealed that the proportion of apoptotic cells was lower in the Farage/R group than in the Farage/S group after treatment with Rituximab (20 μg/mL) (Fig. 1B, C). In addition, when cells in the two groups were stimulated with 20 μg/mL Rituximab for 24, 48, and 72 h, cells in the Farage/R group formed greater numbers of colonies than cells in the Farage/S group (Fig. 1D). The complement system consists of 3 recognition units (C1q, C1r, and C1s), 3 activation units (C2, C3, and C4), and 5 membrane attack units (C5, C6, C7, C8, and C9; Fig. 1E). In the complement activation pathways, C3 is cleaved into C3a and C3b. We found that complement C3 expression was increased in the Farage/R group when compared with the Farage/S, group, while the expression of C3 degradation products showed the reverse trend (Fig. 1F), indicating inhibition of the complement system in Farage/R cells. Moreover, among the 3 recognition units, C1q was downregulated in Farage/R cells when compared to Farage/S cells, while there was no significant difference in C1r and C1s expression between the two groups (Fig. 1G, H). This suggests that the complement activation pathway was restrained via inhibition of C1q in the Farage/R cells.

### C1qA was the key recognition unit for complement system function in Rituximab-resistant cells and was downregulated by m^6^A modification

**Fig. 2:**
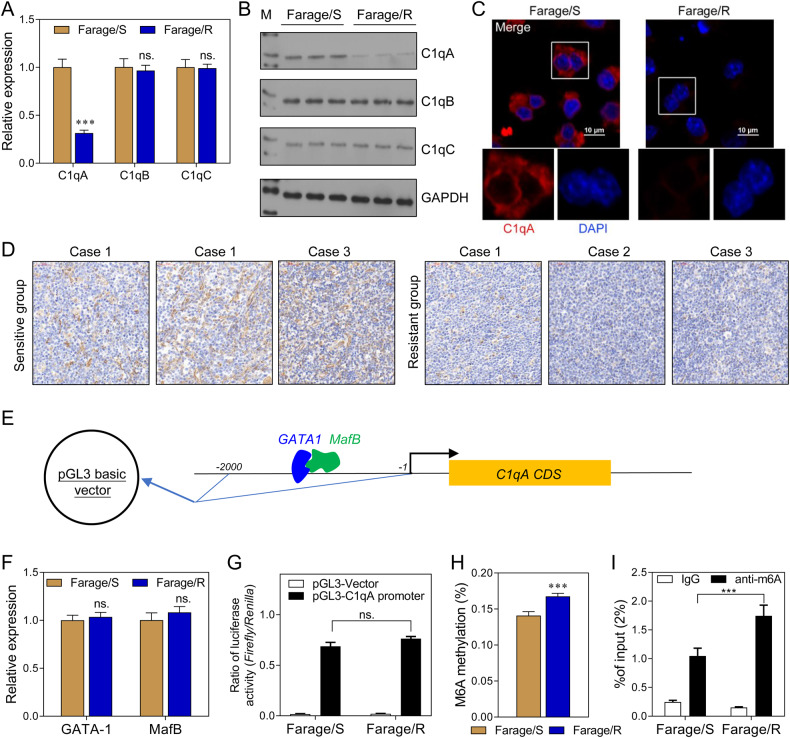
C1qA was the key recognition unit of the complement system related to Rituximab resistance and might be regulated by m^6^A modification. **A** The mRNA levels of 3 subunits of C1q in Farage/S and Farage/R cells were detected by qPCR. **B** The levels of C1qA, C1qB, and C1qC proteins were detected by western blotting. **C** C1qA expression was localized by immunofluorescence. **D** The expressions of C1qA in DLBCL tissues from the first surgery of three pairs of rituximab sensitive and rituximab-resistant patients were showed by IHC assay. **E** A schematic diagram of the C1qA promoter inserted into the luciferase expression vector. **F** The relative expression levels of 2 transcriptional regulators, GATA-1 and MafB, were detected by qPCR. **G** The ratios of luciferase activity in the Farage/S and Farage/R groups. **H** The level of total m^6^A-modified RNA. **I** The level of m^6^A-modified C1qA mRNA as detected by Me-RIP qPCR. ns, not significant; ***p < 0.001.

We further investigated the expression of 3 C1q subunits (C1qA, C1qB, and C1qC). Western blot and immunofluorescence assays showed that C1qA expression was downregulated at both mRNA and protein levels in Farage/R cells, while the other subunits were not affected (Fig. 2A–C). Furthermore, DLBCL tissue samples from the first surgery of three pairs of rituximab-sensitive and rituximab-resistant patients were collected. Results of IHC assay showed that the C1qA expression is different between the two groups (Fig. 2D). Next, we explored the mechanisms that regulate the C1qA expression. GATA-1 and MafB have been reported to be transcriptional regulators of C1qA (Fig. 2E). However, a qPCR analysis showed there was no significant difference in GATA-1/ MafB expression between the Farage/S and Farage/R cells (Fig. 2F). Next, the promoter was inserted into a pGL3 basic vector and the plasmid was transfected into Farage/S and Farage/R cells (Fig. 2E). However, there was no significant difference in luciferase activity between the Farage/S and Farage/R cells (Fig. 2G). These results indicated that C1qA expression was regulated by some other mechanisms, and not GATA-1/MafB. Therefore, we investigated whether the m^6^A modification participated in regulating C1qA expression. The result showed that the levels of both total m^6^A methylation and m^6^A modified C1qA were significantly increased in the Farage/R group when compared to the Farage/S group (Fig. 2H, I). This suggested that C1qA expression might be regulated by m^6^A methylation.

### C1qA reduced the Rituximab resistance of Farage cells

**Fig. 3:**
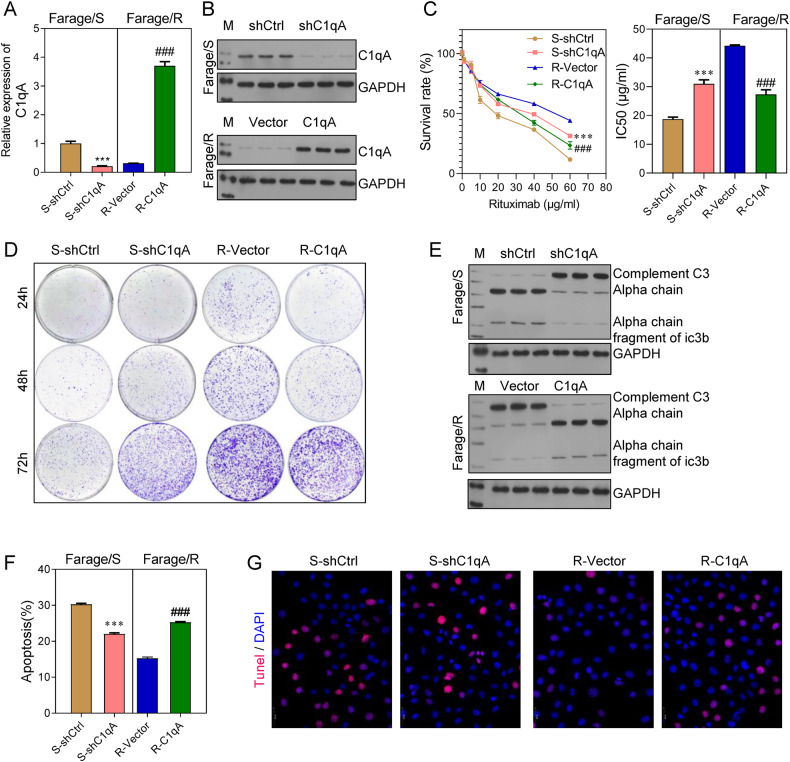
Knockdown of C1qA increased the Rituximab resistance of Farage/S cells, and C1qA overexpression reduced the Rituximab resistance of Farage/R cells. Farage/S cells were transfected with shC1qA and Farage/S cells were transfected with pcDNA 3.0-C1qA. **A**, **B** The expression of C1qA in 4 groups. **C** The survival rates of cells treated with different concentrations of Rituximab, and the IC50 value. **D** The proliferation of cells treated with 20 μg/mL Rituximab was detected by the colony formation assay. **E** C3 chains expressed during treatment with 20 μg/mL Rituximab were detected by western blotting. **F**, **G** Apoptotic cells during treatment with 20 μg/ mL Rituximab were detected by flow cytometry and TUNEL staining. S-shCtrl, Farage/S cells transfected with shcontrol; S-shC1qA, Farage/S cells transfected with shC1qA; R-Vector, Farage/R cells transfected with an empty vector; R-C1qA, Farage/R cells transfected with the C1qA overexpression vector. ***p < 0.001, ###*p* < 0.001.

To confirm the role played by C1qA in the Rituximab resistance of DLBCL, the effects of C1qA knockdown in Farage/S cells and C1qA overexpression in Farage/R cells were investigated (Fig. 3A, B). CCK-8 assays showed that C1qA knockdown increased the survival rate and IC50 values of Farage/S cells, and C1qA overexpression decreased the survival rate and IC50 values of Farage/R cells (Fig. 3C). In addition, colony formation assays indicated that C1qA deficiency promoted the proliferative ability of Farage/S cells, and C1qA overexpression inhibited that promoting effect in Farage/R cells (Fig. 3D). Moreover, C1qA knockdown/overexpression had the reverse effect on cell apoptosis (Fig. 3F, G). Finally, western blot studies revealed that the complement system was suppressed by knockdown of C1qA in Farage/S cells, and stimulated by overexpression of C1qA in Farage/R cells (Fig. 3E).

**Fig. 4:**
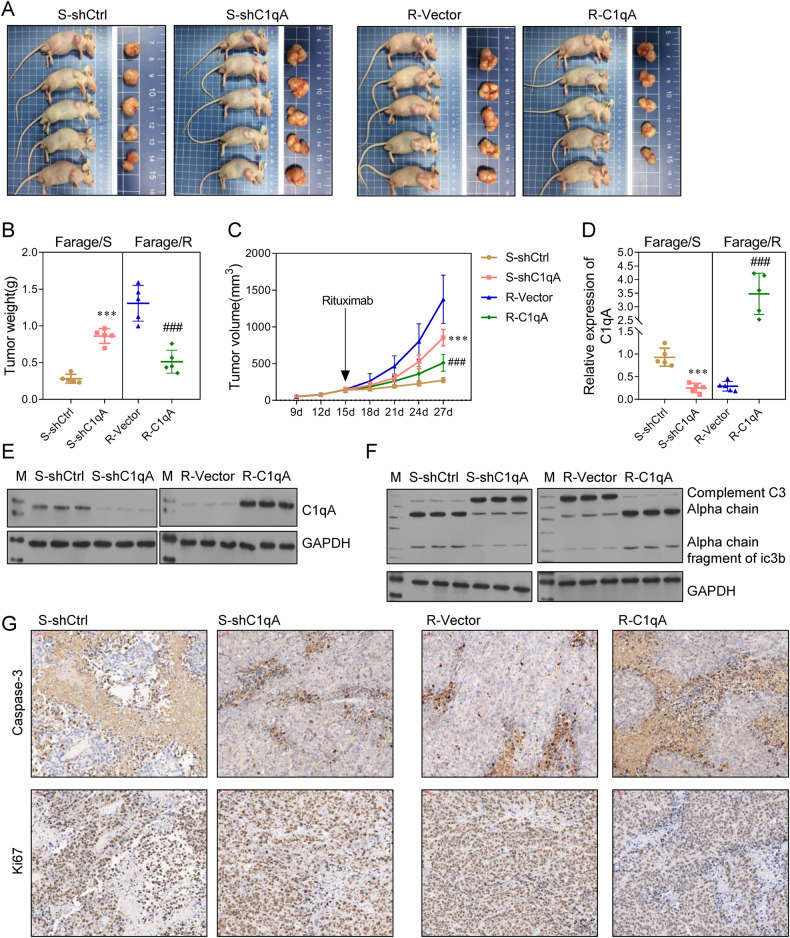
C1qA increased the Rituximab sensitivity of Farage cells in vivo. **A** Mice were subcutaneously injected with S-shCtrl, S-shC1qA, R-Vector, or R-C1qA cells and then treated with Rituximab. On day 27 after xenograft, the mice were sacrificed and the tumor tissues were collected. **B** The weights of the collected tumors. **C** Tumor growth curves were plotted with the tumor size measured every 4 days. **D**, **E** The expression of C1qA in tumor tissues. **F** C3 chains detected by western blotting. **G** Caspase-3 and Ki67 expression in tumor tissue was detected by immunohistochemistry. ***p < 0.001, ### *p* < 0.001.

Our in vitro findings suggested that C1qA could enhance the Rituximab sensitivity of DLBCL cells. To further verify the relationships among C1qA, tumor growth, and the Rituximab resistance of DLBCL, Farage/S cells transfected with shCtrl (S-shCtrl), Farage/S cells transfected with shC1qA (S-shC1qA), Farage/R cells transfected with empty vector (R-Vector), and Farage/R cells transfected with a C1qA overexpression vector (R-C1qA) were used to generate mouse xenograft models. At 15 d after subcutaneous injection of those cells, the mice were treated with Rituximab. The tumors in the S-shC1qA group were larger than those in the S-shCtrl group, and the tumors in the R-C1qA group were smaller than those in the R-Vector group (Fig. 4A–C). In addition, when compared to the S-shCtrl group, the levels of C1qA expression and complement system proteins in tumor tissues from the S-shC1qA group were suppressed. When compared to the R-Vector group, the levels of C1qA expression and the complement system proteins in tumor tissues from the R-C1qA group were increased (Fig. 4D–F). Finally, an IHC analysis of Caspase-3 and Ki67 expression revealed that cell apoptosis was inhibited in the S-shC1qA group when compared with the S-shCtrl group, and increased in the R-C1qA group when compared with the R-Vector group, while assays for cell proliferation showed the opposite results (Fig. 4G).

Taken together, these findings demonstrated that C1qA expression was associated with the Rituximab sensitivity of DLBCL cells. Furthermore, upregulation of C1qA expression promoted the apoptosis of DLBCL cells both in vitro and in vivo.

### METTL3 and YTHDF2 participated in the m^6^A modification of C1qA

**Fig. 5:**
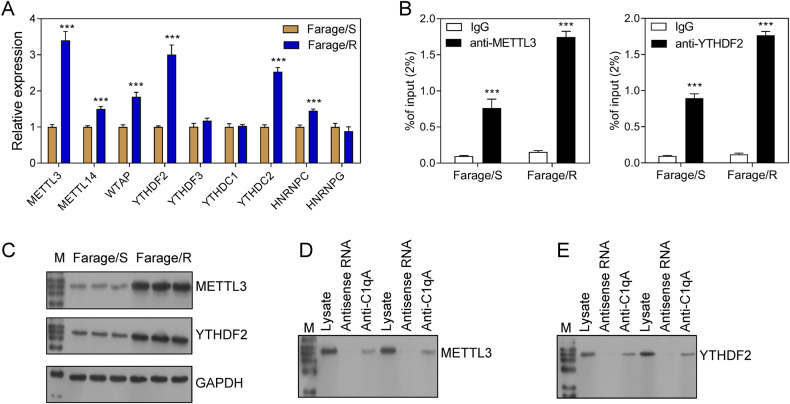
METTL3 and YTHDF2 were bound to C1qA mRNA. **A** Relative expression of readers, writers, and erasers in Farage/S and Farage/R cells as detected by qPCR. **B** C1qA mRNA bound by METTL3 or YTHDF2 was detected by RIP-qPCR. **C** The levels of METTL3 and YTHDF2 in Farage/S and Farage/R cells. **D**, **E** METTL3 and YTHDF2 binding on C1qA mRNA was detected by pull-down-western blotting. ***p < 0.001.

The above-mentioned results suggested that C1qA expression might be regulated by m^6^A methylation. Therefore, experiments were performed to determine whether and how m^6^A methylation regulated C1qA expression. The expression levels of common readers and writers were detected by qPCR, and the results showed that METTL3, METTL14, WTAP, YTHDF2, YTHDC2, and HNRNPC were up-regulated in Farage/R cells when compared with Farage/S cells (Fig. 5A). The MethyTranscriptome DataBase Version 2.0 prediction tool predicted that METTL3 and YTHDF2 were the writer and reader of C1qA methylation, respectively. Moreover, the levels of METTL3 and YTHDF2 proteins were also higher in Farage/R cells than in Farage/S cells (Fig. 5C). RNA immunoprecipitation and qPCR (RIP-qPCR) and pull-down-western blot analyses showed that C1qA was bound by both METTL3 and YTHDF2 (Fig. 5B, D, E).

### METTL3 was a writer for C1qA methylation that inhibited the complement pathway and promoted tumor growth

**Fig. 6:**
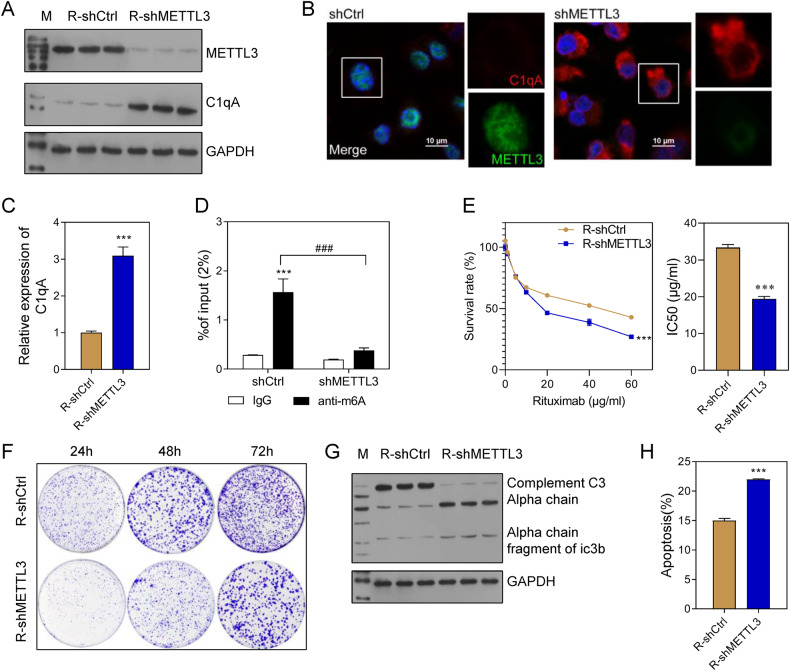
Knockdown of METTL3 increased the Rituximab sensitivity of Farage/R cells. **A**–**C** METTL3 and C1qA expression in Farage/R cells with METTL3 knockdown (R-shMETTL3) and in control cells (R-shCtrl). **D** The levels of m^6^A-modified C1qA. **E** Cell survival rates and the IC50 value of Rituximab. **F** Cell proliferation under conditions of treatment with 20 μg/mL Rituximab was detected by the colony formation assay. **G** C3 chains expressed during treatment with 20 μg/mL Rituximab were detected by western blotting. **H** The proportions of apoptotic cells during treatment with 20 μg/mL Rituximab were detected by flow cytometry. ***p < 0.001, ### *p* < 0.001.

To further confirm the relationship between METTL3 and C1qA, METTL3 expression was knocked down in Farage/R cells. Subsequent results showed that METTL3 was downregulated while C1qA was up-regulated in the R-shMETTL3 group when compared with the R-shCtrl group (Fig. 6A–C). Meanwhile, the levels of m^6^A-modified C1qA in the R-shMETTL3 group were higher than those in the R-shCtrl group (Fig. 6D). In addition, the cell survival rate, IC50 value of Rituximab, and the cell proliferation rate were all decreased in the R-shMETTL3 group (Fig. 6E, F). The ratio of apoptotic cell had the oppositeresult in R-shMETTL3 group (Fig. 6H). Moreover, METTL3 knockdown also stimulated the complement system in Farage/R cells (Fig. 6G).

**Fig. 7:**
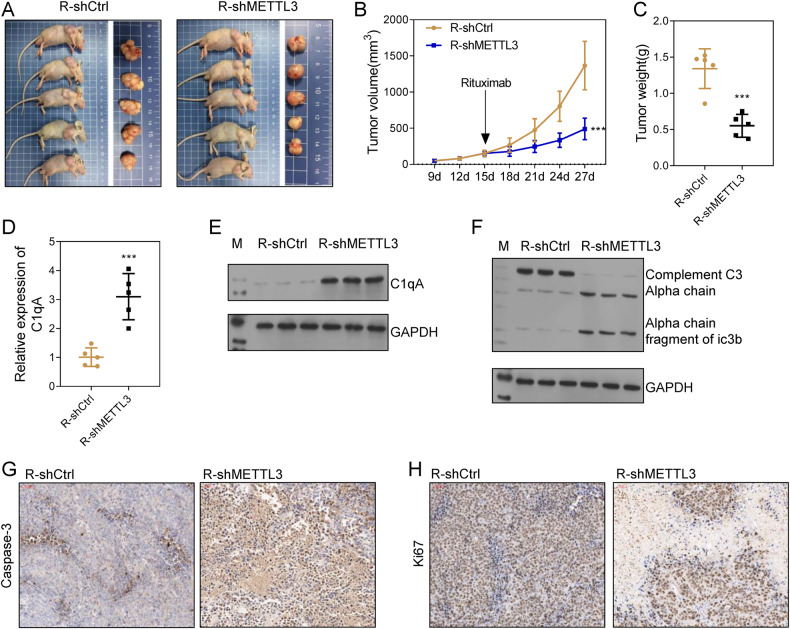
Knockdown of METTL3 increased the Rituximab sensitivity of Farage/R cells in vivo. **A** Mice were subcutaneously injected with R-shCtrl or R-shMETTL3 cells and then treated with Rituximab. On day 27 after xenograft, the mice were sacrificed and the tumor tissues were collected. **B** Tumor growth curves were plotted with the tumor size measured every 4 days. **C** The weights of the collected tumors. **D**, **E** The expression of C1qA in tumor tissues. **F** C3 chains were detected by western blotting. **G**, **H** Caspase-3 and Ki67 expression in tumor tissue was detected by immunohistochemistry. ***p < 0.001.

The role of METTL3 was further demonstrated in vivo. C1qA-deficient Farage/R or control cells were subcutaneously implanted into mice to generate xenograft models. Subsequent measurements showed that tumor growth in the R-shMETTL3 group was suppressed when compared to that in the R-shCtrl group (Fig. 7A–C). In addition, C1qA expression at both the RNA and protein level was up-regulated in the R-shMETTL3 group (Fig. 7D, E). An IHC analysis of Caspase-3 and Ki67 revealed that cell apoptosis was inhibited, and cell proliferation was promoted in tumor tissues from the R-shCtrl group (Fig. 7G, H). Furthermore, western blot analyses indicated that complement system activity in the R-shMETTL3 group was higher than that in the R-shCtrl group (Fig. 7F). Collectively, these findings indicated that METTL3 was a writer for C1qA methylation that inhibited the activity of the complement system and thus promoted tumor cell proliferation both in vitro and in vivo.

### Interference with YTHDF2 increased activity of the complement pathway and the Rituximab sensitivity of Rituximab-resistant DLBCL cells

**Fig. 8:**
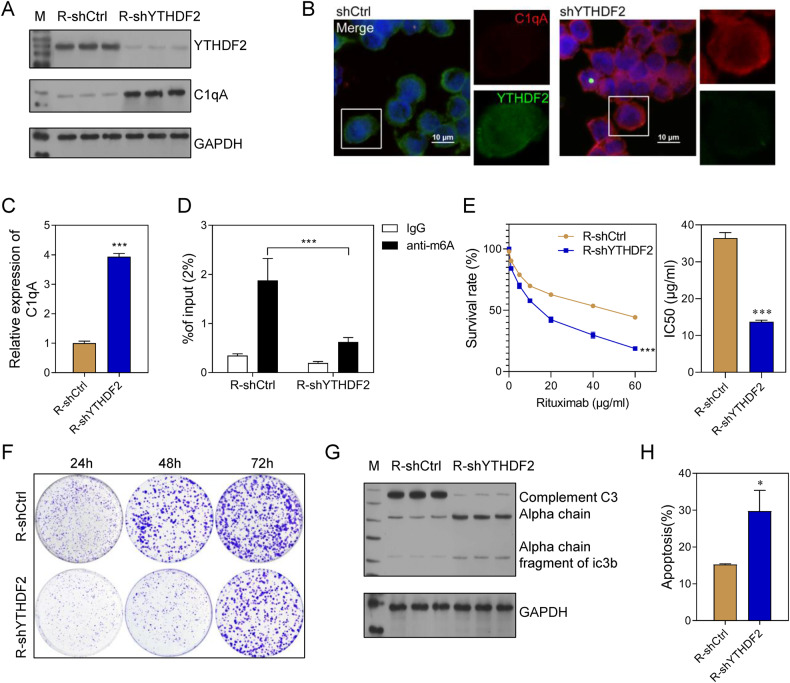
Knockdown of YTHDF2 increased the Rituximab sensitivity of Farage/R cells. **A**–**C** The expression of YTHDF2 and C1qA in Farage/R cells with YTHDF2 knockdown (R-shYTHDF2) and in control cells (R-shCtrl). **D** The level of m^6^A-modified C1qA. **E** Cell survival rates and the IC50 value of Rituximab. **F** The proliferation of cells being treated with 20 μg/mL Rituximab was detected by the colony formation assay. **G** C3 chains expressed during treatment with 20 μg/mL Rituximab were detected by western blotting. **H** The proportions of apoptotic cells during treatment with 20 μg/mL Rituximab were detected by flow cytometry. ***p < 0.001.

To further confirm the function of YTHDF2 and the relationship between YTHDF2 and C1qA, YTHDF2 was knocked down in Farage/R cells. Subsequent western blot and immunofluorescence analyses showed that decreased YTHDF2 expression was accompanied by increased C1qA expression in Farage/R cells (Fig. 8A–C). In addition, the levels of m^6^A-modified C1qA were downregulated in the R-shYTHDF2 group when compared with the R-shCtrl group (Fig. 8D). Knockdown of YTHDF2 reduced the survival rate of Farage/R cells under of conditions of Rituximab treatment; it also decreased the proliferation, and promoted the apoptosis of Farage/R cells (Fig. 8E, F, H). Moreover, complement system activity was stimulated by YTHDF2 knockdown (Fig. 8G). These results demonstrated that YTHDF2 was the reader for C1qA methylation that inhibited the activity of the complement system and thus reduced the Rituximab sensitivity of Farage/R cells.

## Discussion

The complement system is a vital innate immune system, which can affect acquired immunity in humans. A disordered complement system has been detected in various cancers, and is related to drug resistance. Rituximab is an antibody used for DLBCL immunotherapy, and has shown promising results in treatment of DLBCL patients. In addition, the efficacy of Rituximab is dependent on the complement system [10]. However, it remains unclear how the complement system affects the Rituximab resistance of DLBCL. In this study, we found that C1qA was the key factor that affected the Rituximab resistance of DLBCL. In addition, we also found that C1qA was regulated by m^6^A methylation with METTL3 and YTHDF2.

Inhibition of the complement system has been proved to block tumor growth, metastasis, and drug resistance in some cancers. In head and neck squamous cell carcinomas (HNSCCs), the use of C3a and C5a receptor signaling inhibitors resulted in T cell conversion and increased tumor growth [20]. Karuturi et al. [21] discovered that C3, C5, C3AR1, and C5AR1 were related to the activation of immune-related oncogenic processes in various cancers. A low level of C3AR1 expression indicated chemotherapy resistance in colorectal and breast cancer, but increased sensitivity to chemotherapy in glioblastoma multiforme (GBM) and ovarian cancer. C3AR1 expression was also correlated with lymphocyte-mediated tumor killing and a tumor’s sensitivity to immunotherapy. A high level of C3 expression suggested a good outcome for immune checkpoint blockade (ICB) therapy targeting PD1, CTLA4, and ACT in melanoma, but a bad outcome for ICB therapy targeting PD1 and PDL1 in GBM and bladder cancer. Rituximab is an ICB molecule that targets CD20. After its initial interaction, Rituximab subsequently interacts with the immune system, including components in the complement pathway. Therefore, Rituximab resistance is not only related to CD20 expression, but also the complement system [22]. Studies have found that tumor cells can block complement activation cascades and inhibit MAC formation via membrane complement regulatory proteins (mCRPs), such as CD46, CD55, and CD59 [10]. We found that complement system activity in Farage/R cells was lower than that in Farage/S cells. Moreover, of all the recognition units of the complement system, only the expression of C1qA was downregulated in Farage/R cells. Subsequent assays showed that C1qA knockdown in Farage/S cells inhibited the complement system, and further promoted cell proliferation and suppressed apoptosis under conditions of Rituximab treatment. In addition, C1qA overexpression in Farage/R cells promoted the complement system, and further suppressed cell proliferation and promoted apoptosis under conditions of Rituximab treatment. Collectively, suppression of the complement system caused by C1qA downregulation was related to Rituximab resistance in Farage cells, and Rituximab resistance was attenuated by overexpression of C1qA.

C1q is not only a key recognition unit of the complement system, but also a bridge between innate immunity and acquired immunity mediated by IgG/IgM. It contains the subunits C1qA, C1qB, and C1qC [23]. Chen et al. [23] found that C1q might be prognostic factor in osteosarcoma. The survival rate of osteosarcoma patients with high levels of C1q expression was higher than that of patients with low C1q expression. C1q was verified to be related to the outcome of Rituximab therapy. Gaetano et al. [24] found no difference in the survival curves of C1qA^-/-^ C57BL/6 mice that had been xenografted with EL4-CD20^+^ cells (a lymphoma cell line of EL4 stably expressing the human CD20 antigen) and did or did not receive treatment with Rituximab. However, in parallel control experiments, wild-type mice were cured by Rituximab. Moreover, polymorphisms in the *C1qA* gene were found to affect the clinical response and duration of response to Rituximab therapy for follicular lymphoma. Racila et al. [25] found that follicular lymphoma patients with A at C1qA[276 A/G] had longer progression-free survival times than patients who were G/G homozygous. Jin et al. [26] reported that DLBCL patients who were homozygous A showed a better overall response, a higher complete response rate, and a longer overall survival time than DLBCL patients who received Rituximab plus cyclophosphamide/doxorubicin/vincristine/prednisone chemotherapy. Some types of cancer cells, such as lung cancer cell lines, have been proven to activate a local complement system [27]. As a discovery in this study, we found that DLBCL cells activated their own complement system. In addition, the complement system could regulate Rituximab resistance. Of note, C1qA may originate from other non-tumor cells in DLBCL tissue. Blood monocytes and tumor-associated macrophages also expressed *C1qA* and may affect tumor cells through it [28]. More research is needed to determine whether exogenous C1qA protein level is affected by Rituximab.

M^6^A methylation plays a vital role in tumorigenesis, tumor progression, and immunotherapy. As a typical reader for m^6^A methylation, METTL3 and the levels of m^6^A-modified mRNA are up-regulated in many types of cancer cells and tumor tissues [29, 30]. M^6^A levels and METTL3 expression in DLBCL cell lines and DLBCL tissues were higher than those in human B lymphocytes and control inflammatory lymph glands, respectively. Moreover, silencing of METTL3 inhibited the proliferation of DLBCL cells [31]. Feng et al. [30] reported that interference with METTL3 led to decreases in NEDD1 mRNA expression and m^6^A methylation levels in DLBCL cells. In natural killer/T-cell lymphoma (NKTCL), a type of non-Hodgkin’s lymphoma, METTL3 was up-regulated when compared to its expression in normal NK cells. This upregulation can further promote the cisplatin resistance of NKTCL cells [29]. In addition, METTL3 was been reported to shape the tumor microenvironment (TME). In breast cancer cells, METTL3 overexpression up-regulated the m^6^A methylation of PD-L1 mRNA and inhibited T-cell infiltration [32]. In our study, we found that METTL3 was also related to the Rituximab resistance of DLCBL cells. METTL3 was up-regulated in Farage/R cells compared to Farage/S cells and knockdown of METTL3 in Farage/R cells up-regulated C1qA expression and increased the Rituximab sensitivity of Farage/R cells. Collectively, our data showed that METTL3 regulated Rituximab sensitivity by controlling the complement system. We also believe that METTL3 may affect the efficacy of Rituximab by reshaping the TME, as suggested in the above studies.

YTHDF2 is a member of the YTH domain protein family and a direct m^6^A reader. It primarily recognizes and binds to sites of m^6^A methylation in the cytoplasm and can accelerate degradation of its target mRNAs. By cooperating with a writer and eraser, the 3 molecules (reader, writer, and eraser) can control the level of m^6^A methylation on RNAs. In most types of cancer, including leukemia, YTHDF2 is up-regulated in tumor tissues when compared to its levels in normal tissues [33]. YTHDF2 acts as an oncogene that can facilitate cell proliferation and inhibit apoptosis via TNF signaling in acute myeloid leukemia [34]. In addition, YTHDF2 was found to correlate with the number of tumor-infiltrating immune cells in lung adenocarcinoma [35]. However, it has been rarely reported whether YTHDF2 can regulate drug sensitivity. In our study, we found that YTHDF2 was up-regulated in Rituximab-resistant DLBCL cells. YTHDF2 knockdown accelerated activation of the complement system, promoted cell apoptosis, and inhibited cell proliferation under conditions of Rituximab treatment. This indicated that YTHDF2 regulates Rituximab resistance in DLBCL cells by suppressing the complement system.

Collectively, the complement system and m^6^A methylation were both reported to participate in TME establishment. Meanwhile, the TME plays an important role in cancer biology, and affects the effectiveness of immunotherapy, including the efficacy of Rituximab. Therefore, the complement system and m^6^A methylation regulate the Rituximab sensitivity of DLBCL cells, not only by shaping the TME, but also inducing CDC.

In conclusion, activation of the complement system via C1qA was related to the Rituximab resistance of DLBCL cells. In addition, C1qA expression was regulated by m^6^A methylation with METTL3 and YTHDF2. These results suggest that the METTL3/ YTHDF2-C1qA axis is a novel mechanism that controls the Rituximab resistance of DLBCL cells.

## Materials and methods

### Cell culture and transfection

A DLBCL cell line, Farage, was purchased from Procell Life Science&Technology Co.,Ltd. (Wuhan, China). Cells were authenticated by STR profiling and tested for mycoplasma contamination. The cells were cultured in RPMI 1640 medium supplemented with 10% fetal bovine serum and 100 μg/mL streptomycin/ penicillin in an incubator with a 5% CO_2_ humidified atmosphere at 37^o^C.

For knockdown and overexpression, lentivirus vectors containing the respective targeting sequences were purchased from IGE biotech (Guangzhou, China), and transfected into cells by using Lipofectamine 3000 (Thermo Fisher, Waltham, MA, USA) according to the manufacturer’s instructions.

### Construction of Rituximab-resistant DLBCL cells

Rituximab-resistant Farage cells were established by using a concentration gradient progressive increase induction method. In brief, the IC50 value of Rituximab for Farage cells was initially determined by a CCK-8 assay; after which, the Farage cells were exposed to low doses of Rituximab below the IC50 value. Next, the cells were exposed to gradually higher doses of Rituximab until they demonstrated stable growth. After 6 months, Rituximab-resistant Farage cells were obtained and designated as Farage/R. The wild-type Farage cells were designated as Farage/S.

### Cell viability assay

Cells in logarithmic growth phase were seeded into 96-well plates. After receiving the corresponding treatment, Cell Counting Kit-8 solution (CCK-8, Beyotime, Shanghai, China) was added to each well, and the plate was incubated for 2 h at 37^o^C. The absorbance (OD) value of each well was read at 450 nm using a microplate reader (BIO-RAD, Hercules, CA, USA), and compared with blank wells. The half-maximal inhibitory concentration (IC50) was calculated used GraphPad Prism 7 software based on the survival curve.

### Colony formation assay

Treated cells were seeded into 6-well plates and cultured in an incubator at 37^o^C for 14 days. Cell colonies were fixed, washed with PBS, fixed with 4% paraformaldehyde, and then stained with 1% crystal violet (Beyotime, China). Finally, the number of colonies was counted under a microscope (Olympus, Tokyo, Japan).

### Cell apoptosis analysis

Apoptotic cells were detected by flow cytometry and the TUNEL assay. For flow cytometry, cells were collected and stained using an Annexin-V/FITC and propidium iodide (PI) detection kit (BD Biosciences, San Jose, USA) according to the manufacturer’s instructions. The stained cells were analyzed by a flow cytometry (BD Bioscience). Cells stained with Annexin V were considered as apoptotic. For the TdT-mediated dUTP nick end labeling (TUNEL) assay, cells were fixed with 4% paraformaldehyde, permeabilized with 0.5% Triton X-100, and then incubated with TUNEL reaction mixture (Roche, Switzerland) at 37^o^C for 1 h in the dark. Signals were observed under a fluorescence microscope (Olympus).

### Western blotting

Proteins were extracted from cells or tissues using RIPA lysis buffer (Thermo Fisher). The protein concentration in each extract was detected using a BCA Protein Assay Kit (Beyotime). A sample of protein from each extract was separated by 10% SDS-PAGE, and the protein bands were transferred onto polyvinylidene fluoride membranes (Millipore, Darmstadt, Germany), which were subsequently blocked with 5% BSA for 2 h. Next, the membranes were incubated for 1 h with primary antibodies against METTL3 (ab195352, 1:1000, Abcam, Cambridge, UK), YTHDF2 (ab246514, 1:1000), C3 (ab200999, 1:2000), C1q (ab11861, 1:2000), C1r (ab190800, 1:3000), C1s (ab134943, 1:2000), C1qA (ab189922, 1:2000), C1qB (ab92508, 1:2000), C1qC (ab75756, 1:1000), and GAPDH (ab8245, 1:10000, Abcam), and then incubated with a secondary antibody (Abcam) for 1 h. The immunostained protein bands were detected using an enhanced chemiluminescence reagent (Thermo Fisher).

### Qualitative PCR

Relative levels of gene expression were detected by qualitative PCR (qPCR). TRIzol reagent (Invitrogen, Carlsbad, CA, USA) was used extract the total RNA from cells and tissue samples. The quality and concentration of RNA in each extract were determined by agarose gel electrophoresis. Reverse transcription was carried out using a PrimeScript™ RT Kit (Takara, Tokyo, Japan). qPCR was performed by using a SYBR green kit (Takara) on an ABI 7500 Real-Time PCR System (Applied Biosystems, Waltham, MA, USA). Relative levels of gene expression were calculated by the 2^−ΔΔCt^ method, with GAPDH serving as an internal control gene. The primers used for qPCR were synthesized by RiboBio (Guangzhou, China), and are shown in Supplementary Table 1 (Table S1).

### Immunofluorescence

Cells were fixed with 4% paraformaldehyde for 20 min, permeabilized with 0.5% Triton X-100 for 30 min, and then blocked with 5% BSA for 1 h. Next, the cells were incubated with anti-C1qA antibody (ab189922, 1:500, Abcam) for 1 h, and subsequently stained with a secondary antibody labeled with Alexa Fluor^TM^ 647 (1:200, ab150075, Abcam). For co-localization of C1qA and METTL3/YTHDF2, the cells received a second round of staining with anti-METTL3/anti-YTHDF2 and a secondary antibody labeled with Alexa Fluor^TM^ 488. The cell nucleus was stained with Diamidino phenyl indole (DAPI; Beyotime). Fluorescence images were captured with a fluorescence microscope (Olympus).

### Detection of m^6^A-modified RNA

First, the level of m^6^A methylation of total RNA was measured by using an EpiQuik m^6^A RNA methylation quantification kit (EpigenTek, Farmingdale, NY USA) according to the manufacturer’s instructions. Next, the m^6^A methylation level of C1qA was detected by MeRIP-qPCR that was performed using a MeRIP m6A Transcriptome profiling Kit (RIBOBIO, Guangzhou, China). Briefly, the total purified mRNA from each sample was fragmented into segments of ~100 nucleotides. These segments were incubated with Magnetic Beads conjugated with anti-m6A antibody. mRNA molecules with m^6^A methylation were eluted from the beads, and their relative expression levels were determined by qPCR.

### Immunohistochemistry

The paraffin blocks containing cancer or adjacent tissues obtained from three rituximab-resistant patients and three rituximab-sensitive patients in the initial surgery were collected. The paraffin blocks were used in detecting the expression level of C1qA, and the process was approved by the Medical Ethics Committee of the Second Affiliated Hospital of South China University of Technology (No. 20200323). In addition, samples of tumor tissue obtained from xenograft model were fixed with 4% paraformaldehyde, dehydrated with an increasing gradient alcohol series plus xylene, embedded in paraffin, and cut into 4-µm-thick sections. The sections were then dewaxed and re-hydrated with xylene plus a decreasing gradient alcohol series. Next, the sections were blocked with 5% FBS, incubated overnight with primary anti-Caspase 3 (ab32351, 1:100), anti-Ki67 antibodies (ab16667, 1:200), or anti-C1qA (ab189922, 1:500) and then incubated with a secondary antibody for 1 h. Signals were detected by diaminobenzidine (DAB) staining and cell nuclei were stained with hematoxylin. Finally, the sections were mounted in neutral resin and observed under a microscope (Olympus).

### RNA pull-down

The proteins bound to C1qA mRNA were detected by RNA pull-down assays that were performed using a Pierce^TM^ Magnetic RNA-Protein Pull-Down Kit (Thermo Fisher). Briefly, C1qA mRNA sequences synthesized by Guangzhou IGE Biotech (China) were labeled with biotinylated cytidine bisphosphate. The labeled RNAs were incubated with streptavidin magnetic beads, and the beads were incubated with the corresponding cell lysates. Finally, the proteins on the beads were eluted and subsequently detected by western blotting

### RNA immunoprecipitation and qPCR

The RIP assay was performed by using an RNA Immunoprecipitation Kit (BersinBio, Guangzhou, China) according to the manufacture’s instruments. Briefly, cells were lysed in polysome lysis buffer containing a protease inhibitor, RNase inhibitor, and DNase. The lysate was collected by centrifugation and divided into 3 parts (one for IP, one for IgG, and one for Input). Anti-C1qA/IgG and protein A/G beads were added to the lysate. Co-precipitated RNAs were extracted from the beads. Finally, the level of m^6^A-modified C1qA was detected by qPCR.

### Xenograft model

Male BALB/c nude mice (*n* = 30; age = 5 weeks) were obtained from the Experimental Animal Center of Sun Yat sen University (Guangzhou, China) and randomly divide into three groups. Approximately 2 × 10^6^ Farage/R or Farage/S cells stably transfected with shNC, shC1qA or shMETTL3 were subcutaneously injected into the left flank of each mouse. When the tumor volume reached ~100 mm^3^, each mouse received an intraperitoneal injection of Rituximab (20 mg/kg) every 4 days for a total of 5 injections. The diameter of each tumor was examined every 4 days using a caliper, and tumor volume was calculated as follows: (length × width^2^)/2. At 28 days after xenograft, the mice were sacrificed and the tumors were weighed and collected. The investigator was blinded to the groupallocation during the experiment. The experiment was approved by Experimental Animal Ethics Committee of South China University of Technology (No. 20200320).

### Statistical analysis

All statistical data were analyzed using GraphPad Prism 7 software, and results are presented as a mean value ± standard deviation. The unpaired two-tailed Student’s t test or/and one-way ANOVA with Turkey’s post hoc test were used to analyze differences between two or multiple groups. A *P*-value < 0.05 was considered to be statistically significant.

### Supplementary information

Supplementary Table 1

Cell lines and report

Original Data File

## Data Availability

Data can be provided upon reasonable request.
