# Supplementary material for: METTL3-mediated m6A methylation of C1qA regulates the Rituximab resistance of diffuse large B-cell lymphoma cells
Source: Cell Death Discov. 2023 Nov 1;9:405. doi: 10.1038/s41420-023-01698-2 (PMC10618261; doi:10.1038/s41420-023-01698-2)
Supplement: Supplementary file 1 — Supplementary Table 1 [file 41420_2023_1698_MOESM1_ESM.docx]

Supplementary Table 1 The primers used for qPCR

| Name | Squence |
| --- | --- |
| GAPDH F | TGTTCGTCATGGGTGTGAAC |
| GAPDH R | ATGGCATGGACTGTGGTCAT |
| C1qA F | GCCCAGGAAACATCAAGGAC |
| C1qA R | GGAGTGGTTCTGGTACGGTT |
| C1qC F | CCAACCCGCAGGGAGATTATG |
| C1qC R | CCGAGTTGACCTGATTGGTTTT |
| GATA-1 F | TTGTCAGTAAACGGGCAGGTA |
| GATA-1 R | CTTGCGGTTTCGAGTCTGAAT |
| MafB F | TCAAGTTCGACGTGAAGAAGG |
| MafB R | GTTCATCTGCTGGTAGTTGCT |
| METTL3 F | GAACACAGAGCTTAAATCCCCA |
| METTL3R | TGTCAGCTAAACCTACATCCCTG |
| METTL14 F | TCAAGTTCGACGTGAAGAAGG |
| METTL14 R | GTTCATCTGCTGGTAGTTGCT |
| WTAP F | CTTCCCAAGAAGGTTCGATTGA |
| WTAP R | TCAGACTCTCTTAGGCCAGTTAC |
| YTHDF2 F | CTAGCTCCTTAGGTGGAGCC |
| YTHDF2 R | TTGCTTGCAACTTCTGTGCT |
| YTHDF3 F | TCAGAGTAACAGCTATCCACCA |
| YTHDF3 R | GGTTGTCAGATATGGCATAGGCT |
| YTHDC1 F | AACTGGTTTCTAAGCCACTGAGC |
| YTHDC1 R | GGAGGCACTACTTGATAGACGA |
| YTHDC2 F | CAAAACATGCTGTTAGGAGCCT |
| YTHDC2 R | CCACTTGTCTTGCTCATTTCCC |
| HNRNPC F | GGCTTTGCCTTCGTTCAGTA |
| HNRNPC R | GGACGGAGAAGGGTGTTCTG |
| HNRNPG F | ACTCAAGTGGTCGTGATCGG |
| HNRNPG R | ATCAGATCGGCTTCCTCCAC |
