# Supplementary figures and images for: METTL3-mediated m6A methylation of C1qA regulates the Rituximab resistance of diffuse large B-cell lymphoma cells
Source: Cell Death Discov. 2023 Nov 1;9:405. doi: 10.1038/s41420-023-01698-2 (PMC10618261; doi:10.1038/s41420-023-01698-2)

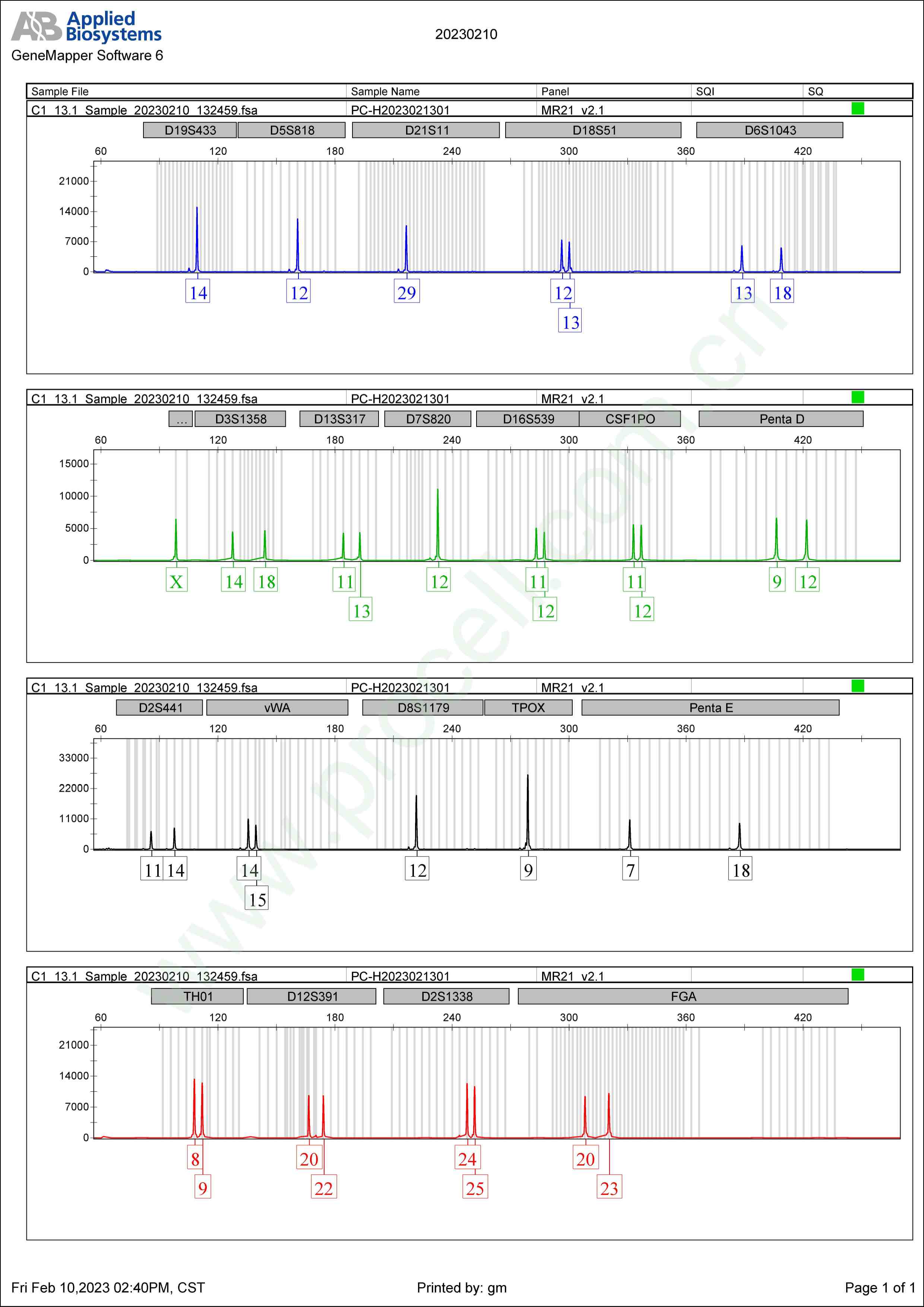

Supplement: Supplementary file 2 — Cell lines and report [file 41420_2023_1698_MOESM2_ESM.jpg]

Figure 1

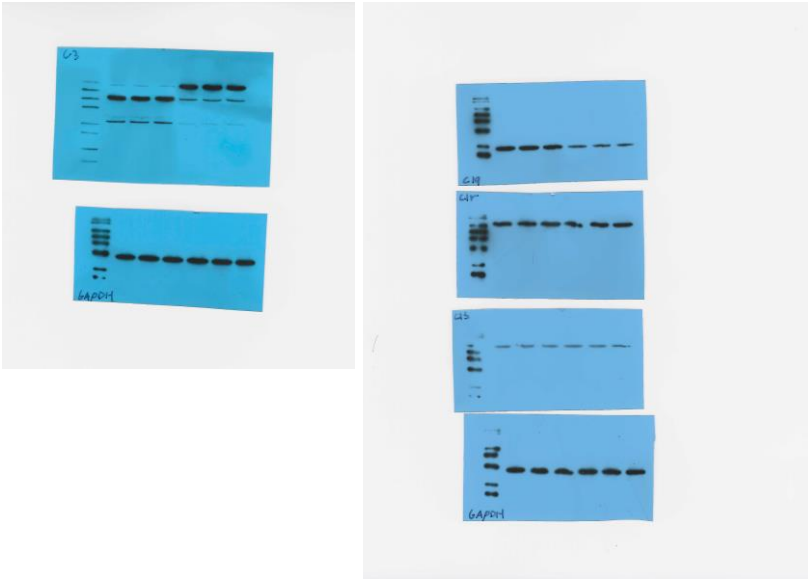

Figure 2

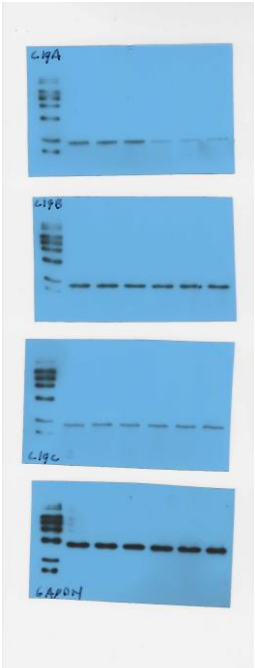

Figure 3

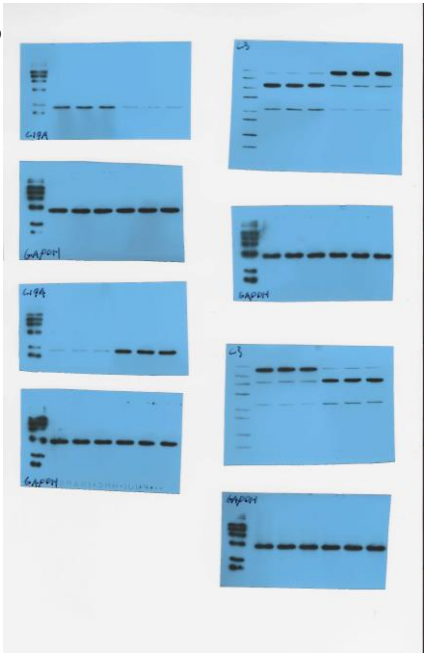

Figure 4

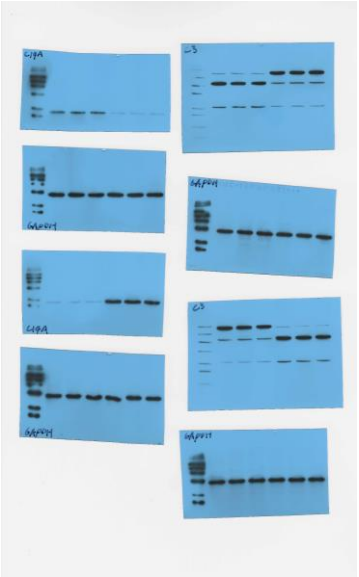

Figure 5

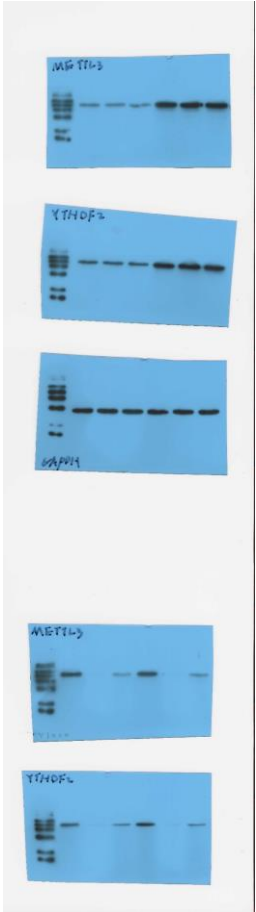

Figure 6

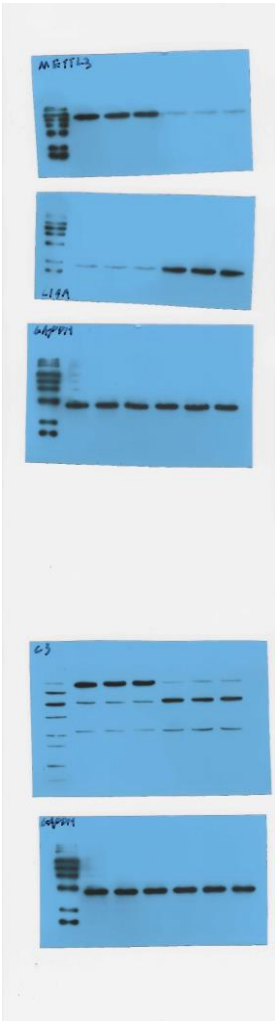

Figure 7

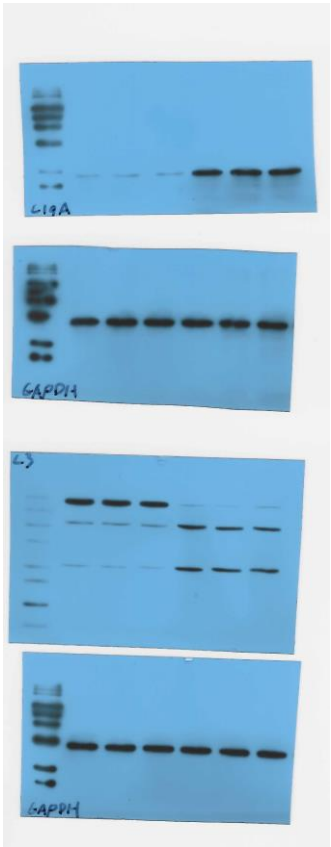

Figure 8

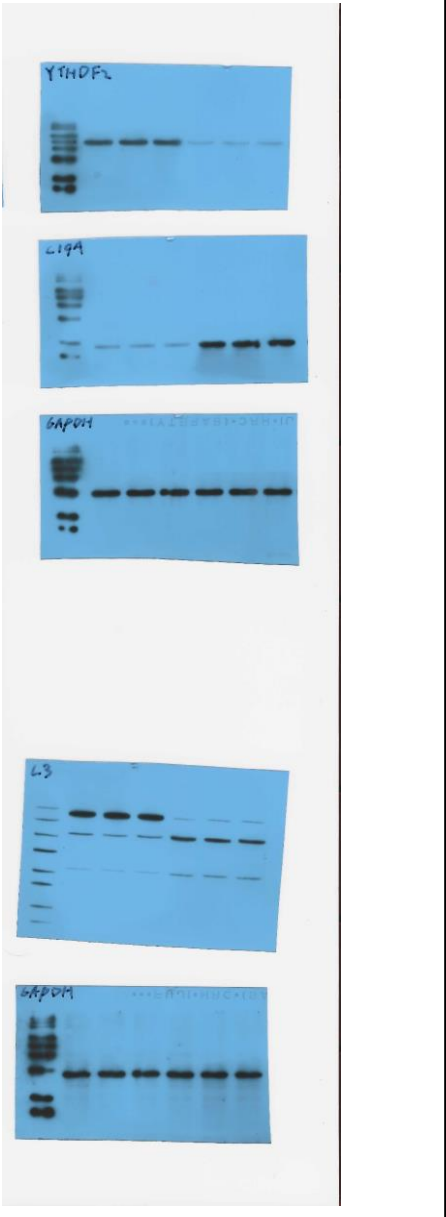

Supplement: Supplementary file 3 — Original Data File [file 41420_2023_1698_MOESM3_ESM.pdf]
